# A Time-Series Metabolomic Analysis of SARS-CoV-2 Infection in a Ferret Model

**DOI:** 10.3390/metabo12111151

**Published:** 2022-11-21

**Authors:** Avinash V. Karpe, Thao V. Nguyen, Rohan M. Shah, Gough G. Au, Alexander J. McAuley, Glenn A. Marsh, Sarah Riddell, Seshadri S. Vasan, David J. Beale

**Affiliations:** 1 Land and Water, Commonwealth Scientific and Industrial Research Organisation, Ecosciences Precinct, Dutton Park, QLD 4102, Australia; 2 Department of Chemistry and Biotechnology, School of Science, Computing and Engineering Technologies, Swinburne University of Technology, Hawthorn, VIC 3122, Australia; 3 Commonwealth Scientific and Industrial Research Organisation, Australian Centre for Disease Preparedness, Geelong, VIC 3220, Australia; 4 Department of Health, 189 Royal Street, East Perth, WA 6004, Australia; 5 Department of Health Sciences, University of York, York YO10 5DD, UK

**Keywords:** COVID19, SARS-CoV-2, metabolomics, omics, animal models, ferret, host metabolic responses

## Abstract

The global threat of COVID-19 has led to an increased use of metabolomics to study SARS-CoV-2 infections in animals and humans. In spite of these efforts, however, understanding the metabolome of SARS-CoV-2 during an infection remains difficult and incomplete. In this study, metabolic responses to a SAS-CoV-2 challenge experiment were studied in nasal washes collected from an asymptomatic ferret model (*n* = 20) at different time points before and after infection using an LC-MS-based metabolomics approach. A multivariate analysis of the nasal wash metabolome data revealed several statistically significant features. Despite no effects of sex or interaction between sex and time on the time course of SARS-CoV-2 infection, 16 metabolites were significantly different at all time points post-infection. Among these altered metabolites, the relative abundance of taurine was elevated post-infection, which could be an indication of hepatotoxicity, while the accumulation of sialic acids could indicate SARS-CoV-2 invasion. Enrichment analysis identified several pathways influenced by SARS-CoV-2 infection. Of these, sugar, glycan, and amino acid metabolisms were the key altered pathways in the upper respiratory channel during infection. These findings provide some new insights into the progression of SARS-CoV-2 infection in ferrets at the metabolic level, which could be useful for the development of early clinical diagnosis tools and new or repurposed drug therapies.

## 1. Introduction

Highly transmissible and pathogenic coronavirus (CoV) infections are well-established sources of epidemics in humans and have caused global concerns. The severe acute respiratory syndrome CoV (SARS-CoV) first emerged in Guangdong, China, in 2002 [1]. The SARS-CoV virus rapidly spread to 29 countries and caused 8096 infections and 774 deaths worldwide, according to the World Health Organization (WHO) [2]. The Middle East respiratory syndrome CoV (MERS-CoV) was first recorded in Saudi Arabia in 2012 and has rapidly spread to 27 countries [3]. As of May 2022, the total MERS-CoV infections reported globally was 2591 including 894 deaths, according to the WHO [4]. The novel coronavirus disease, COVID-19, caused by SARS-CoV-2 was first reported in Wuhan, China, and has rapidly spread worldwide causing over 522 million cases [5]. Although the mortality rate is relatively low (1.2%), with 5.94 million deaths reported between 1 January 2020 and 31 December 2021, analysis of excess mortality for this two-year period has led to an estimate of 17.1–19.6 million deaths at 95% uncertainty interval [6]. Current estimates are 6.5 million deaths recorded [5] and up to 26.5 million excess deaths globally [7].

The COVID-19 etiology is progressively being unraveled, but the underlying molecular mechanisms and the associated metabolic alterations remain poorly understood. The COVID-19 infection reflects a broad spectrum of patient symptoms. Several pathophysiological pathways are perturbed during the progression of disease. This complexity led us to investigate this exciting topic using metabolomics. Metabolomics studies the changes in holistic endogenous metabolites, presenting essential insights into a cell’s metabolic state in response to physiological and pathological disturbances. Harnessing these metabolic outputs can potentially lead to the discovery of signature metabolic biomarkers relevant to pathogenesis. Such metabolic biomarkers could then be applied to personalized medicine, the development of public healthcare strategies, and/or the designing of Point-of-Care (PoC) testing regimens for more rapid testing [8,9].

Most metabolomics studies related to COVID-19 have focused on the identification of new potential biomarkers of the disease. For example, Wu, et al. [10] reported altered energy metabolism (due to reduced malic acid of the TCA cycle) and hepatic dysfunction (due to reduced carbamoyl phosphate of the urea cycle and D-xylulose-5-phosphate of the pentose phosphate pathway). Barberis, et al. [11] suggested that monolaurin could have a potential defensive role against SARS-CoV-2 infection and demonstrated that people with higher cholesterol levels are at a higher risk of developing SARS-CoV-2 infection. The dysregulation of macrophage, platelet degranulation, complement system pathways, and massive metabolic suppression have been reported in COVID-19 patient sera [12].

The dynamic shift of metabolic pathways is exhibited by living organisms to cope with various perturbations. Monitoring the dynamic metabolic changes during disease development has attracted increasing interest in recent years. Evidence from the literature suggests that the metabolomics studies based on time-series data could possibly provide insight into the interfacial stage between normal and diseased states and further facilitate the screening of biomarkers for early diagnosis. For example, Villoslada, et al. [13] studied the metabolomic signatures associated with disease severity in multiple sclerosis. Sphingomyelin and lysophosphatidylethanolamine were identified as putative biomarkers in the time series analysis for discriminating between multiple sclerosis patients and healthy individuals [13]. Jacyna, et al. [14] investigated the urine metabolic profiles of bladder cancer patients pre- and post-resection. It was reported that hippuric acid, pentanedioic acid and uridine could potentially be used for sample differentiation [14]. Huang, et al. [15] analyzed the time-series lipidomics data to study the development of hepatocellular carcinoma in a hepatocarcinogenesis rat model. A ratio of lysophosphatidylcholine 18:1/free fatty acid (FFA) 20:5 was identified as the potential biomarker for hepatocellular carcinoma [15]. Li, et al. [16] used time-series metabolomics to study progressive stages of cholestatic liver fibrosis in a mouse model. Taurocholic acid, tauromuricholic acid, lysophosphatidylethanolamine 20:2, sulfoglycolithocholic acid, and taurohyodeoxycholic acid were associated with the progression of the hepatocyte injury index, and docosahexaenoic acid, arachidonic acid, proline, leucine, and linoleic acid were associated with the progression of liver fibrosis index, liver hydroxyproline [16]. The metabolic profile during progression of cardiac heart failure was studied to discover potentially new biomarkers of the disease [17]. Twenty-three metabolites were altered in the rat model of myocardial infarction-induced cardiac heart failure. The branched-chain amino acids, leucine and valine, were found to differentiate between the rat failing hearts and healthy hearts [17].

In our previous study, we analyzed nasal wash samples from SARS-CoV-2-infected ferrets [18]. Multivariate analysis of the acquired data identified 29 significant metabolites and three significant lipids in the nasal wash samples. The presence of viral shedding coincided with the challenge dose administered and significant changes in the citric acid cycle, purine metabolism, and pentose phosphate pathways, amongst others, in the nasal wash samples [18]. In the current study, time-series metabolomics data from a SARS-CoV-2-infected ferret model were analyzed to provide insights into the viral progression and metabolic responses of ferrets to the virus infection. In addition, the study aimed to identify sex-specific responses of male and female ferrets following SARS-CoV-2 exposure. For that purpose, ten outbred male and ten outbred female ferrets (*Mustela putorius furo*) were challenged with SARS-CoV-2 via the intranasal route [19], and nasal wash samples were collected for metabolomics analysis at six time points (3 days pre-infection and 3, 5, 7,9 and 14 days post-infection).

## 2. Materials and Methods

### 2.1. Ferret Challenge and Sample Collection

Ten outbred male and ten outbred female ferrets (*Mustela putorius furo, n* = 20) at the age of 4 months were used for this challenge experiment. The selection of ferrets was based on previously reported observations using this model [20,21,22,23]. The study was reviewed by the Animal Ethics Committee (AEC) at the Australian Centre for Disease Preparedness (ACDP) (AEC 1990). All animal work in this study was conducted in a PC-4 containment facility at the ADCP in Geelong, Australia. Animal housing, husbandry, and handling for sample collections were as previously described [24]. Ferrets were acclimatized in cages at the facility for 7 days prior the experiment. During this period, animals were monitored daily and given food and water ad libitum, and environmental enrichment. Before the challenge, ferrets were implanted with a LifeChip Bio-Thermo transponder (Destron Fearing, Dallas, TX, USA); subcutaneous temperature, rectal temperature and body weight were recorded.

SARS-CoV-2 (BetaCoV/Australia/VIC01/2020) was used for the challenge experiment which was provided by the Victorian Infectious Diseases Reference Laboratory (The Peter Doherty Institute, Melbourne, VIC, Australia) [25]. The viral preparation was conducted as the method described by Au et al. [19]. All ferrets were challenged with SARS-CoV-2 VIC01 via the intranasal route (0.5 mL total volume diluted in PBS) at a target dose of approximately 9 × 10^4^ TCID_50_ (back-titered to 4.64 × 10^4^ TCID_50_). The inoculum was back-titrated by TCID_50_ assay on Vero E6 cells to confirm the administered dose.

Following the virus challenge, ferrets were monitored daily for the presence of clinical signs (e.g., reduced-interaction score, fever, sneezing, coughing and respiratory disease). Animals were anesthetized for collection of nasal wash samples, as well as for the measurement of rectal temperature and body weight on days 3, 5, 7, 9 and 14 post virus-challenge. All samples were rapidly frozen, and then gamma-irradiated (50 kGy) to inactivate the SARS-CoV-2 virus for safe removal of samples from the PC-4 containment laboratory. All samples were stored at −80 °C before extraction and metabolomics analysis. Reverse-transcription qPCR was performed as per Marsh, et al. [21].

### 2.2. Metabolomics Analysis

The metabolite extraction was carried out as previously described [18]. The frozen nasal wash samples (100 µL) were thawed and extracted with 450 µL of ice-cold methanol and ethanol solution (1:1, *v*/*v*) and 100 µL milliQ water (Merck, Darmstadt, Germany). Samples were vortexed at 2000 rpm for 10 min before centrifugation at 20,000× *g* for 2 min at 4 °C. The metabolite and lipid extracts were separated via the Captiva EMR-Lipid plate (2 mL, Agilent, Mulgrave, VIC, Australia). A rinse of 200 µL Water: Methanol: Ethanol (2:1:1) through the same Captiva EMR-Lipid tube was performed. The extracted solution was dried under nitrogen stream, followed by the resuspension in 50 µL of 20% methanol (in water). The resuspended samples were re-vortexed at 1000 rpm at room temperature for 45 min and analyzed on an Agilent 6470 Liquid Chromatography Triple Quadrupole Mass Spectrometer (LC-QqQ-MS) (Agilent Technologies, Mulgrave, VIC, Australia) for central carbon metabolite (CCM) analysis. Furthermore, discovery metabolites (non-CCM) were analyzed using the same suspension on an Agilent 6546 Liquid Chromatography Time-of-Flight Mass Spectrometer (LC-QToF) with an Agilent Jet Stream source coupled to an Agilent Infinity II UHPLC system (Agilent Technologies, Santa Clara, CA, USA). The analysis was performed as per the previous method [18]. A series of blanks, mixed QC standards were prepared in the same way. Pooled biological quality control (PBQC) samples were prepared by combining 5 µL aliquots from each biological sample. Internal standards of 1 ppm L-phenylalanine (1-^13^C) and succinic acid (1,4-^13^C_2_) were used. The residual relative standard deviation (RDS%) of the internal standards were 8.98% (L-Phenylalanine, 1-^13^C) and 6.54% (Succinic Acid, 1,4-^13^C_2_).

### 2.3. Statistical Analysis and Data Integration

Identification of metabolites from acquired data was conducted using MassHunter Quantitative Analysis Software and Profinder (v0B.10.0: Agilent Technologies, Santa Clara, CA, USA). The acquired CCM data were first subtracted by blanks, then normalized to internal standards (L-phenylalanine ^13^C and succinic acid ^13^C). Untargeted metabolite data were normalized to reference ions (positive mode =; negative mode =). The metabolomics data were subjected to further statistical analysis using multivariate statistics. The data were first imported, matched by sample identifiers (metadata), and log-transformed to normalize the data using SIMCA 16.02 (MKS Data Analytics Solutions, Uméa, Sweden). Partial least square-discriminant analysis (PLS-DA) was performed by finding successive orthogonal components from the SARS-CoV-2 isolate and sample type-specific datasets with maximum squared covariance and was subsequently used to identify the common relationships among the multiple datasets. All models were cross-validated using CV-ANOVA in SIMCA, which is a diagnostic approach for assessing the reliability of PLS and OPLS models.

MetaboAnalyst 5.0 (Xia Lab, McGill University, Montreal, QC, Canada) was also used for the univariate and multivariate analysis, biomarker analysis, enrichment and metabolic pathway analysis [26]. Metabolite features with >50% missing values were excluded. A Log_10_ normalisation and auto-scaling were applied to the filtered metabolite features. Metabolites with a Benjamini–Hochberg adjusted *p*-value of ≤ 0.05 were considered to be statistically significant [27]. Chemical clusters based on structural similarity were created for metabolic examination using the ChemRICH analysis [28].

## 3. Results and Discussions

No clinical signs and perturbations on bodyweight were observed in ferrets after the virus challenge. However, an increase of viral RNA shedding, and changes of metabolites were recorded in the nasal wash, indicating the infection of the virus in ferrets. The upper respiratory tract is one of the primary sites of SARS-CoV-2 infection [23,29]. The current study involved the nasal wash as the representative of the site of the nasal tract to assess biochemical changes during SARS-CoV-2 infection in the ferret model.

### 3.1. Viral Shedding following Challenge

**Figure 1:**
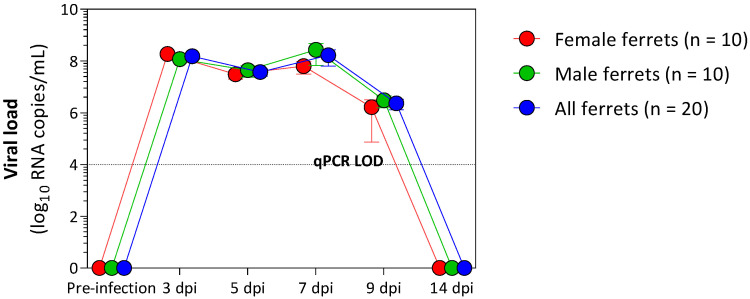
Viral RNA shedding in nasal washes collected from SARS-CoV-2-infected ferrets (*n* = 20). Red and green dots represent female (*n* = 10) and male ferrets (*n* = 10), respectively, while all ferrets (both males and females) are annotated in blue. SARS-CoV-2 RNA was detected in nasal wash samples from ferrets at 3, 5, 7, and 9 dpi. The dotted line represents the limit of detection (LOD) of the reverse-transcription qPCR assay.

Viral RNA was detected in the nasal wash of 20 ferrets from 3 days post-infection (dpi) and continued to be detected at varying levels until 9 dpi (Figure 1). The peak in viral RNA shedding was seen at 3 dpi and 7 dpi for all ferrets. A decline in viral RNA was seen at 5 dpi and 9 dpi for all ferrets. The viral RNA declined below the limit of quantification of the assay at 14 dpi, at which point no viral RNA was detected in their nasal washes. Similar observations on viral RNA shedding have been reported in the ferret model [19,23,30]. In humans, the mean time of viral shedding was reported to be 14 days [30,31]. However, there is a wide variability of SARS-CoV-2 shedding among studies, reflecting the heterogeneity of human populations [32,33,34].

### 3.2. Central Carbon Metabolism Variance in the Nasal Wash Samples

The nasal washes from the infected ferrets were subjected to a central carbon metabolism metabolite screening via a LC-QqQ-MS method. The samples indicated the presence of 82 out of the 223 common polar metabolites from the central carbon metabolism and related pathways. The residual relative standard deviation (RSD%) of the internal standards was 8.99% (L-phenylalanine 1-^13^C) and 6.54% (succinic acid 1,4-^13^C_2_). The RSD% of the QC standards (Supplementary Table S1) was <10% with the exception of lactic acid (RSD% = 11.07%). Within the PBQC samples, a total of 34 metabolites indicated an RSD% of <10% (Supplementary Table S2).

**Figure 2:**
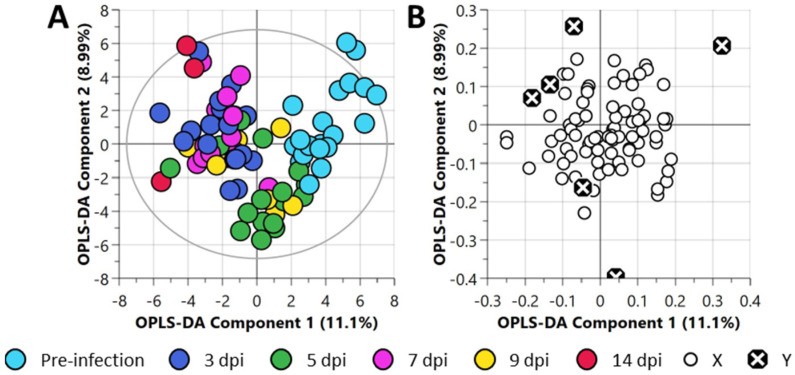
Orthogonal partial least square-discriminant analysis (OPLS-DA) of the central carbon metabolism metabolite dataset of nasal wash samples collected from ferrets. (**A**) OPLS-DA scatter plot and (**B**) OPLS-DA loadings plot. For this plot, R^2^X (cum) = 0.453, R^2^Y (cum) = 0.304, Q^2^ = 0.108. The ellipse presented in panel (**A**) represents Hotelling’s T2 confidence limit (95%). The colored circles in panel (**A**) represent each analyzed sample, while the black crossed circles in panel (**B**) indicate the average group position for each sample cluster, with the white circles representing the distribution of metabolite features between these groups.

This dataset was processed via an unsupervised statistical approach using Principal Component Analysis (PCA) (Supplementary Figure S1). Any sub-data clustering was not evident from the PCA analysis. The grouped data were then analyzed using a supervised partial least square-discriminant analysis (PLS-DA) (Supplementary Figure S2) and orthogonal PLS-DA (OPLS-DA) (Figure 2) to explore metabolic differences in infected ferrets as a function of time. The PLS-DA analysis of nasal wash samples did not yield any better sub-data clustering. Whilst the PLS-DA dataset was found to be statistically non-significant (*p*-value > 0.05) when cross-validated (Supplementary Table S3), the OPLS-DA model dataset was found to be statistically significant (*p*-value < 0.05, Supplementary Table S4).

### 3.3. Chemical and Pathway Analysis of the Central Carbon Metabolism

The chemical analysis of the entire central carbon metabolism dataset indicated that monosaccharides, tricarboxylic acids (TCA), benzoic acids, organic dicarboxylic acids, fatty acids and conjugates, disaccharides, purines, amino acids and peptides, pyridines, carboxylic acids, pyrimidines, sulfonic acids, hydroxy acids, delta valerolactones, phosphate esters, keto acids, and benzamides were significantly (*p*-value < 0.05) enriched chemical classes.

**Figure 3:**
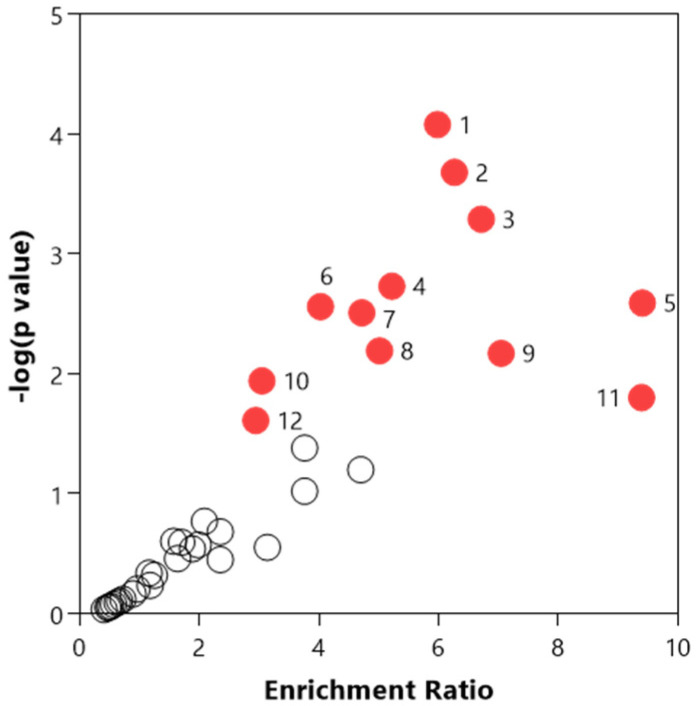
Relevant pathways identified using the central carbon metabolism dataset in nasal washes collected from ferrets. The red-colored circles represent significantly relevant pathways, while the white circles represent the non-significant pathways. Noting, 1: Pentose phosphate pathway, 2: Pentose and glucuronate interconversions, 3: Arginine biosynthesis, 4: Starch and sucrose metabolism, 5: D-Glutamine and D-glutamate metabolism, 6: Alanine, aspartate and glutamate metabolism, 7: Citrate cycle (TCA cycle), 8: Butanoate metabolism, 9: Valine, leucine and isoleucine biosynthesis, 10: Amino sugar and nucleotide sugar metabolism, 11: Phenylalanine, tyrosine and tryptophan biosynthesis, and 12: Glyoxylate and dicarboxylate metabolism.

**Figure 4:**
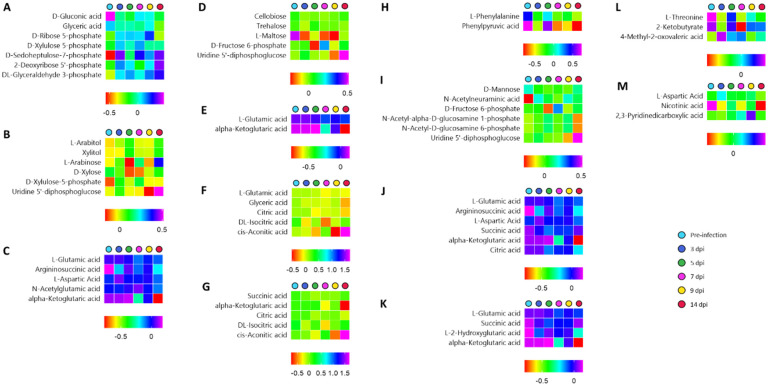
Time-series observed for metabolites related to the 13 significantly relevant central carbon metabolic pathways. (**A**) Pentose phosphate pathway, (**B**) Pentose and glucuronate interconversions, (**C**) arginine biosynthesis, (**D**) starch and sucrose metabolism, (**E**) D-glutamine and D-glutamate metabolism, (**F**) glyoxylate and dicarboxylate metabolism, (**G**) citrate cycle, (**H**) phenylalanine, tyrosine and tryptophan biosynthesis, (**I**) amino sugar and nucleotide sugar metabolism, (**J**) alanine, aspartate and glutamate metabolism, (**K**) butanoate metabolism, (**L**) valine, leucine and isoleucine biosynthesis, and (**M**) nicotinate and nicotinamide metabolism.

The entire central carbon metabolism dataset was used to identify the most relevant pathways (Supplementary Table S5). Several pathways including the pentose phosphate pathway, pentose and glucuronate interconversions, arginine biosynthesis, starch, and sucrose metabolism, D-glutamine and D-glutamate metabolism, alanine, aspartate and glutamate metabolism, citrate cycle, butanoate metabolism, valine, leucine, and isoleucine biosynthesis, amino sugar and nucleotide sugar metabolism, phenylalanine, tyrosine, and tryptophan biosynthesis, glyoxylate and dicarboxylate metabolism and nicotinate and nicotinamide metabolism were found to be significantly relevant (*p*-value < 0.05, Figure 3). Figure 4 illustrates the time-series observed for the central carbon metabolites contributing to these pathways.

In our previous study, pentose phosphate pathway, purine metabolism and citrate cycle were identified to be the key metabolite pathways in the ferret nasal cavity during SARS-CoV-2 infection [18]. The current study indicated that the sugar and glycan metabolism pathways leading from and to the non-oxidative parts of energy pathways were of key importance in the upper respiratory channel during the SARS-CoV-2 infection. Particularly, in our studies, the pentose phosphate pathway (PPP) has shown to play a key role during SARS-CoV-2 infection. Recently reported proteomic study indicated that the enzymes such as transketolase (TKT) and transaldolase 1 (TALDO1), which contribute towards the non-oxidative part of PPP, were upregulated in the cells infected with SARS-CoV-2 [35]. Due to this phenomenon, biochemicals such as 2-deoxy glucose (2DG) and benfooxythiamine (BOT), which inhibit the non-oxidative pathways of PPP, have shown to inhibit SARS-CoV-2 replication [35,36]. In addition to this, recent correlation network analysis in patients with COVID-19 has also shown that in addition to the viral infection, the upper respiratory microbiota also gets affected during SARS-CoV-2, which in turn has been shown to be correlated with the upregulation of pathways such as PPP [37]. Although we did not study the impact of SARS-CoV-2 infection on nasal microbial population and metabolism, a further study of host-microbiome-virus interactomics is expected to shed more light on this biochemical mechanism.

PPP-associated pathways such as glycan metabolism (reflected through the pentose and glucuronate interconversions) and amino sugar and nucleotide sugar metabolism appeared to be key pathways in the ferret nasal cavity during SARS-CoV-2 infection. Some of the early genomic studies have shown that the SARS-CoV-2-triggered surface protein receptors (STSPRs) such as Ephrin type-A receptor 6 (EPHA6) are differentially expressed in the human lungs. The EPHA6 proteins have been indicated to be enriched for pentose and glucuronate interconversions [38]. Our study experimentally confirms the outputs of the bioinformatics-based observations of Forst, et al. [38] that, in the ferret upper respiratory tract, the pentose and glucuronate interconversions generally upregulate, particularly around 5–7 dpi (Figure 4B). It has been shown in poultry studies that infection stress due to high-density stocking elevated the pentose and glucuronate interconversion pathway, during the upregulated interleukin (IL)-1β and IL-10 activities in the tracheal barrier and plasma, possibly providing immune response against an infection [39]. Furthermore, the treatment regimen involving bleomycin + pirfenidone drug supplementation for treating lung fibrosis in mice indicated an upregulated pentose and glucuronate interconversion pathway. This study indicated the positive role of this metabolic pathway in providing an immune response to the host during respiratory disorders [40]. Thus, our study indicated a likely increased immune response to SARS-CoV-2 infection. However, a further proteomic study would be able to highlight the importance of this pathway in conferring its role towards the host immune response.

Similar to the pentose and glucuronate interconversion pathway, a recent bioinformatics study indicated that the amino sugar pathway expression has been associated with immunity-related pathways upon the expression of SARS-CoV-2 transmembrane serine protease 2 (TMPRSS2) during COVID-19 [41]. This is an important aspect as it has been shown that the blood plasma amino sugars are key parts of the bio-signaling N-acetyl glycoproteins such as GlycA and GlycB and are important in providing the immune response during SARS-CoV2 infection in patients [42]. Furthermore, it has also been observed that glutaminolysis and glycolysis are essential for virus replication during SARS-CoV-2 infection, and inhibiting these pathways is important to counter virus replication [43]. Our observations of depleted glutamine and glutamate pathways (Figure 4E) indicated a possibly elevated immune response in the respiratory tract cells of the ferrets. Our metabolic output confirms the output of these genomics-based studies through metabolic output. Also, the time-series observations indicated an increased immune response by 7 dpi in the ferret model. However, the proteomic studies will be able to provide further confirmation of these observations.

### 3.4. Multivariate Analysis of the Central Carbon Metabolism and Discovery Metabolites

The post hoc ANOVA indicated the presence of 28 significant central carbon metabolism metabolites (FDR-adjusted *p*-value ≤ 0.05) in the nasal washes (Supplementary Table S6). The samples were further analyzed using a Liquid Chromatography Quadrupole Time-of-Flight Mass Spectrometry (LC-QToF-MS) method to identify the discovery metabolites. Eight additional significant metabolic features were identified (Supplementary Table S7). The significant metabolites were used to identify the most relevantly disturbed metabolic pathways due to the viral infection. These significant metabolites were then used to identify the most significantly disturbed metabolic pathways. While the phenylalanine, tyrosine and tryptophan biosynthesis, phenylalanine metabolism, and butanoate metabolism were significantly (*p* < 0.05) enriched and impacted (Supplementary Table S8), other pathways such as amino sugar and nucleotide sugar metabolism and citrate cycle were significantly enriched and impacted, respectively.

### 3.5. Time-Series Metabolomics Analysis of the Progression of SARS-CoV-2 Infection

**Figure 5:**
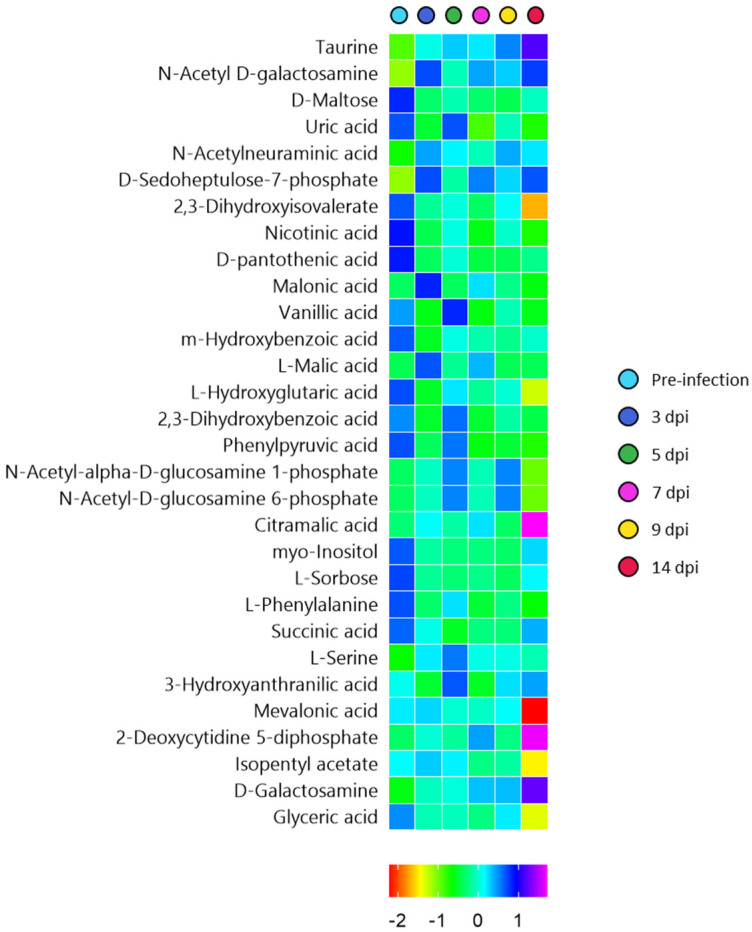
Heat map representing the significant metabolites that were found to significantly vary identified using two-way analysis of variance.

A two-way analysis of variance (ANOVA) was then conducted to decompose the raw data to further determine the contribution of two independent parameters (sex and time) and their interaction. The abundance of several metabolic features was significantly affected by time (Figure 5). No metabolic features were significantly affected by sex or interaction between sex and time.

The thirty significant features included D-sedoheptulose-7-phosphate, N-acetyl D-galactosamine, uric acid, vanillic acid, taurine, phenylpyruvic acid, nicotinic acid, L-2-hydroxyglutaric acid, 2,3-dihydroxyisovalerate, D-pantothenic acid, malonic acid, 2,3-dihydroxybenzoic acid, L-maltose, L-malic acid, N-acetylneuraminic acid, m-hydroxybenzoic acid, N-acetyl-alpha-D-glucosamine 1-phosphate, N-acetyl-D-glucosamine 6-phosphate, citramalic acid, myo-inositol, L-sorbose, L-phenylalanine, succinic acid, L-serine, 3-hydroxyanthranilic acid, mevalonic acid, 2-deoxycytidine 5-diphosphate, isopentyl acetate, D-galactosamine, and glyceric acid (Figure 5).

Of these metabolites, taurine, N-acetyl D-galactosamine, N-acetylneuraminic acid, D-sedoheptulose 7-phosphate, citramalic acid and L-galactosamine increased from pre-infection to 14 dpi. Other metabolites such as L-maltose, 2,3-dihydroxyisovalerate, nicotinic acid, D-pantothenic acid, m-hydroxybenzoic acid, glyceric acid, and 2-hydroxyglutaric acid decreased as the infection progressed. Vanillic acid, uric acid and 2,3-dihydroxybenzoic acid decreased at 3 dpi, showed a slight increase at 5 dpi, and then decreased again. Myo-inositol, L-sorbose, succinic acid and L-phenylalanine decreased post-challenge.

The up- and down-regulation of these metabolites according to infection time could be signatures of viral infection on the host. For example, the elevated taurine could be an indication of hepatotoxicity due to SARS-CoV-2 infection. These observations are consistent with previous studies of SARS-CoV-2, indicating the role of elevated taurine in liver injury, which could be due to a high prevalence of abnormal aminotransferase enzymes [44]. Several members of the CoV family use sialic acids, such as N-acetylneuraminic acid and N-acetyl-D-galactosamine, which are abundantly expressed on the host cell surface of the respiratory tract, as attachment points [45,46]. Elevated sialic acids in the current study indicate SARS-CoV-2 invasion. Particularly high levels of sialic acids were found at 3 dpi and 7 dpi, when the viral load was at the peak. Nicotinic acid with reported anti-inflammatory properties is thought to influence the immune response [44]. The decline in nicotinic acid at 3 dpi and 7 dpi indicates the infectious nature of SARS-CoV-2 represented by high levels of viral load. Reduced levels of pantothenic acid could cause a lack of vitamin B5 and, thus, compromise the mitochondrial energy metabolism [47]. Bruzzone, et al. [48] reported increased levels of phenylalanine and succinic acid in COVID-19 patients. On the contrary, the levels of these metabolites were found to decrease in the current study. Nevertheless, this may all be related to dysregulation of hepatic central carbon metabolism. Li, et al. [49] investigated the use of uric acid as a prognostic marker of COVID-19 patients. Declined levels of uric acid from 7 dpi in nasal samples of ferrets in the current study align well with the observations by Li, et al. [49]. Hence, this metabolite could be used as a marker to assess the severity of COVID-19 for both human and animal models.

## 4. Conclusions

The present study demonstrated the use of a ferret model to study the progression of SARS-CoV-2 infection and the metabolic responses of the host to infection. The viral RNA was detected at 3 dpi and remained detectable until 9 dpi with the absence of clinical signs, indicating that ferrets are an appropriate model for studies of asymptomatic SARS-CoV-2 infection [23]. Along with the change in viral shedding, differences in metabolic responses of the host to different stages of the infection were observed via a time-series metabolomic analysis. This highlighted the power of metabolomics approaches for the systemic characterization of the disease via non-invasive sampling. In addition, this approach provided some metabolite candidates (e.g., uric acid, sialic acids) that could be used as a prognostic indicator of SARS-CoV-2 infection and a biomarker to access the disease severity of COVID-19.

## Data Availability

The data are available on request from the corresponding author. The metabolite data of this study are not publicly available due to animal ethics and intellectual property restrictions.

## Supplementary Materials

The following supporting information can be downloaded at: https://www.mdpi.com/article/10.3390/metabo12111151/s1, Figure S1: Principal component analysis (PCA) of the central carbon metabolism metabolite dataset of nasal wash samples collected from ferrets; Figure S2: Partial least square-discriminant analysis (PLS-DA) of the central carbon metabolism metabolite dataset of nasal wash samples collected from ferrets; Table S1: Composition of QC mix standards (1 ppm) applied to assess the variability of the LC-QQQ-MS metabolomic analysis for central carbon metabolites; Table S2: Composition of PBQC metabolites applied to assess the variability of the LC-QQQ-MS metabolomic analysis for central carbon metabolites; Table S3: Cross-validation (CV)-ANOVA of the PLS-DA metabolomics model (Figure S2); Table S4: Cross-validation (CV)-ANOVA of the OPLS-DA metabolomics model (Figure 2); Table S5: Pathway enrichment analysis of the central carbon metabolic pathways using the central carbon metabolism dataset; Table S6: Multivariate ANOVA analysis of a metabolomics-derived dataset of nasal wash samples collected from ferrets at several time points; Table S7. Significant discovery of metabolites observed through LC-QTOF-MS analysis. Table S8: Pathway enrichment and impact analysis of a metabolomics-derived dataset of nasal wash samples collected from ferrets.
