# Supplementary material for: A Time-Series Metabolomic Analysis of SARS-CoV-2 Infection in a Ferret Model
Source: Metabolites. 2022 Nov 21;12(11):1151. doi: 10.3390/metabo12111151 (PMC9699618; doi:10.3390/metabo12111151)
Supplement: Supplementary file 1 [file metabolites-12-01151-s001.zip › metabolites-1989748-supplementary.pdf]

Supplementary Materials

Table S1. Composition of QC mix standards (1 ppm) applied to assess the variability of the LC-QQQ-MS metabolomic analysis for central carbon metabolites.

QC Standard	RSD (%)
L-Arginine	6.11
L-Histidine	9.58
L-Proline	5.96
L-Serine	4.36
L-Cystine	7.39
L-Threonine	3.73
L-Homoserine	6.79
L-Methionine	4.76
L-Isoleucine	5.29
L-Leucine	4.51
L-Tyrosine	8.21
L-Glutamic acid	5.48
L-Phenylalanine	4.00
L-Aspartic Acid	3.33
Lactic acid	11.07
Succinic acid	4.40

Table S2. Composition of PBQC metabolites applied to assess the variability of the LC-QQQ-MS metabolomic analysis for central carbon metabolites.

PBQC metabolite	RSD (%)
L-Glutamine	8.23
4-Guanidobutyric acid	8.64
L-Tyrosine	7.85
Pyridoxal hydrochloride	8.75
Inosine	7.68
2-Deoxyinosine	5.78
L-Kynurenine	8.06
Guanosine	5.83
L-Glutamic acid	2.14
L-Phenylalanine	2.68
L-Aspartic Acid	2.30
Uric acid	9.28
Thymidine	7.15
D-Gluconic acid	4.32
Galactonic acid	4.21
L-Dihydroorotic acid	4.07
N-Acetyl-alpha-D-glucosamine 1-phosphate	8.82
N-Acetyl-D-glucosamine 6-phosphate	8.82
D-Sedoheptulose-7-phosphate	5.14
Nicotinic acid	9.29
4-Hydroxybenzoic acid	8.49
D-pantothenic acid	7.99
Maleic acid	9.66
Malonic acid	7.69
Succinic acid	1.77

PBQC metabolite	RSD (%)
Vanillic acid	9.72
m-Hydroxybenzoic acid	6.63
L-Malic acid	5.71
L-Hydroxyglutaric acid	6.12
N-Acetylglutamic acid	8.07
Isopentyl acetate	6.36
4-Hydroxyphenyl-pyruvic acid	8.81
D-Fructose 1,6-biphosphate	5.78
4-Pyridoxic acid	8.40

Note: Only metabolite features with RSDs ≤ 10% are represented in the table.

Table S3. Cross-validation (CV)-ANOVA of the PLS-DA metabolomics model (**Figure S2**)

PLS-DA (Figure S2)	SS	DF	MS	F-statistic	p-value	SD
Total corr.	400	400	1			1
Regression	24.3018	20	1.21509	1.229	0.226342	1.10231
Residual	375.698	380	0.98868			0.994324

In this table, SS: sum-of-squares, DF: degrees of freedom, MS: mean squares, SD: standard deviation

Table S4. Cross-validation (CV)-ANOVA of the OPLS-DA metabolomics model (**Figure 2**)

PLS-DA (Figure 2)	SS	DF	MS	F-statistic	p-value	SD
Total corr.	400	400	1			1
Regression	60.0163	40	1.50041	1.58874	0.0158789	1.22491
Residual	339.984	360	0.944399			0.971802

In this table, SS: sum-of-squares, DF: degrees of freedom, MS: mean squares, SD: standard deviation

Table S5. Pathway enrichment analysis of the central carbon metabolic pathways using the central carbon metabolism dataset.

Metabolic pathway	Total	Expected	Hits	Raw *p*-value	Holm *p*-value	FDR	Enrichment ratio
Pentose phosphate pathway	22	1.17	7	<0.0001	0.00699	0.00699	5.982906
Pentose and glucuronate interconversions	18	0.958	6	0.0002	0.0174	0.00881	6.263048
Arginine biosynthesis	14	0.745	5	0.0005	0.0424	0.0145	6.711409
Starch and sucrose metabolism	18	0.958	5	0.0019	0.151	0.0373	5.219207
D-Glutamine and D-glutamate metabolism	6	0.319	3	0.0026	0.207	0.0373	9.404389
Alanine, aspartate and glutamate metabolism	28	1.49	6	0.0028	0.219	0.0373	4.026846
Citrate cycle (TCA cycle)	20	1.06	5	0.0031	0.243	0.0373	4.716981
Butanoate metabolism	15	0.798	4	0.0065	0.499	0.0625	5.012531
Valine, leucine and isoleucine biosynthesis	8	0.426	3	0.0067	0.509	0.0625	7.042254
Amino sugar and nucleotide sugar metabolism	37	1.97	6	0.0116	0.872	0.0977	3.045685
Phenylalanine, tyrosine and tryptophan biosynthesis	4	0.213	2	0.0157	1	0.12	9.389671
Glyoxylate and dicarboxylate metabolism	32	1.7	5	0.0244	1	0.171	2.941176
Nicotinate and nicotinamide metabolism	15	0.798	3	0.0416	1	0.269	3.759398
Ascorbate and aldarate metabolism	8	0.426	2	0.0636	1	0.381	4.694836
Phenylalanine metabolism	10	0.532	2	0.0954	1	0.534	3.759398
Galactose metabolism	27	1.44	3	0.1700	1	0.894	2.083333
Histidine metabolism	16	0.852	2	0.2080	1	1	2.347418
Aminoacyl-tRNA biosynthesis	48	2.55	4	0.2500	1	1	1.568627
Glycine, serine and threonine metabolism	33	1.76	3	0.2550	1	1	1.704545
Pantothenate and CoA biosynthesis	19	1.01	2	0.2680	1	1	1.980198
Nitrogen metabolism	6	0.319	1	0.2800	1	1	3.134796

Metabolic pathway	Total	Expecte d	Hits	Raw *p*-value	Holm *p*-value	FDR	Enrichment ratio
Fructose and mannose metabolism	20	1.06	2	0.2890	1	1	1.886792
Propanoate metabolism	23	1.22	2	0.3490	1	1	1.639344
Taurine and hypotaurine metabolism	8	0.426	1	0.3550	1	1	2.347418
Purine metabolism	65	3.46	4	0.4590	1	1	1.156069
Inositol phosphate metabolism	30	1.6	2	0.4810	1	1	1.25
Glycerolipid metabolism	16	0.852	1	0.5850	1	1	1.173709
Pyrimidine metabolism	39	2.08	2	0.6250	1	1	0.961538
beta-Alanine metabolism	21	1.12	1	0.6850	1	1	0.892857
Sphingolipid metabolism	21	1.12	1	0.6850	1	1	0.892857
Glycolysis / Gluconeogenesis	26	1.38	1	0.7620	1	1	0.724638
Glutathione metabolism	28	1.49	1	0.7870	1	1	0.671141
Phosphatidylinositol signaling system	28	1.49	1	0.7870	1	1	0.671141
Porphyrin and chlorophyll metabolism	30	1.6	1	0.8090	1	1	0.625
Cysteine and methionine metabolism	33	1.76	1	0.8390	1	1	0.568182
Glycerophospholipid metabolism	36	1.92	1	0.8640	1	1	0.520833
Arginine and proline metabolism	38	2.02	1	0.8780	1	1	0.49505
Valine, leucine and isoleucine degradation	40	2.13	1	0.8910	1	1	0.469484
Tryptophan metabolism	41	2.18	1	0.8970	1	1	0.458716
Primary bile acid biosynthesis	46	2.45	1	0.9220	1	1	0.408163

Table S6. Multivariate ANOVA analysis of a metabolomics-derived dataset of nasal wash samples collected from ferrets at several time-points.

Metabolite feature	KEGG ID	F-statistic	FDR	Fisher's LSD
D-Sedoheptulose-7-P	C05382	14.607	3.59E-08	14 dpi > Pre; 3 dpi > 5 dpi; 3 dpi > Pre; 7 dpi > 5 dpi; 5 dpi > Pre; 7 dpi > Pre; 9 dpi > Pre
N-Acetyl D-galactosamine	C01132	14.241	3.59E-08	14 dpi > 5 dpi; 14 dpi > Pre; 3 dpi > 5 dpi; 3 dpi > Pre; 5 dpi > Pre; 7 dpi > Pre; 9 dpi > Pre
Uric acid	C00366	10.425	3.55E-06	5 dpi > 14 dpi; Pre > 14 dpi; 5 dpi > 3 dpi; Pre > 3 dpi; 5 dpi > 7 dpi; 5 dpi > 9 dpi; 9 dpi > 7 dpi; Pre > 7 dpi; Pre > 9 dpi
Vanillic acid	C06672	7.475	0.000172	5 dpi > 14 dpi; Pre > 14 dpi; 5 dpi > 3 dpi; Pre > 3 dpi; 5 dpi > 7 dpi; 5 dpi > 9 dpi; Pre > 7 dpi
Taurine	C00245	7.438	0.000172	14 dpi > 3 dpi; 14 dpi > 7 dpi; 14 dpi > Pre; 3 dpi > Pre; 5 dpi > Pre; 7 dpi > Pre; 9 dpi > Pre
Phenylpyruvic acid	C00166	7.318	0.000172	5 dpi > 14 dpi; Pre > 14 dpi; 5 dpi > 3 dpi; Pre > 3 dpi; 5 dpi > 7 dpi; 5 dpi > 9 dpi; Pre > 7 dpi; Pre > 9 dpi
Nicotinic acid	C00253	6.868	0.000299	Pre > 14 dpi; Pre > 3 dpi; Pre > 5 dpi; Pre > 7 dpi; Pre > 9 dpi
L-2-Hydroxyglutaric acid	C02630	5.869	0.001277	5 dpi > 14 dpi; 7 dpi > 14 dpi; 9 dpi > 14 dpi; Pre > 14 dpi; 5 dpi > 3 dpi; Pre > 3 dpi; Pre > 7 dpi; Pre > 9 dpi
2,3-Dihydroxyisovalerate	C04039	5.806	0.001277	3 dpi > 14 dpi; 5 dpi > 14 dpi; 7 dpi > 14 dpi; 9 dpi > 14 dpi; Pre > 14 dpi; Pre > 3 dpi; Pre > 5 dpi; Pre > 7 dpi
Mevalonic acid	C00418	5.724	0.001315	3 dpi > 14 dpi; 5 dpi > 14 dpi; 7 dpi > 14 dpi; 9 dpi > 14 dpi; Pre > 14 dpi
D-pantothenic acid	C00864	5.506	0.001709	Pre > 14 dpi; Pre > 3 dpi; Pre > 5 dpi; Pre > 7 dpi; Pre > 9 dpi
Malonic acid	C00383	4.888	0.004381	3 dpi > 14 dpi; 3 dpi > 5 dpi; 3 dpi > 9 dpi; 3 dpi > Pre
2,3-Dihydroxybenzoic acid	C00196	4.766	0.004961	5 dpi > 14 dpi; 5 dpi > 3 dpi; Pre > 3 dpi; 5 dpi > 7 dpi; 5 dpi > 9 dpi; Pre > 7 dpi
L-Maltose	C00208	4.435	0.008063	Pre > 3 dpi; Pre > 5 dpi; Pre > 7 dpi; Pre > 9 dpi
L-Phenylalanine	C00079	4.149	0.012247	Pre > 14 dpi; Pre > 3 dpi; 5 dpi > 7 dpi; Pre > 7 dpi; Pre > 9 dpi
2-Deoxycytidine 5'-diP	C00705	4.018	0.014351	14 dpi > 3 dpi; 14 dpi > 5 dpi; 14 dpi > 7 dpi; 14 dpi > 9 dpi; 14 dpi > Pre; 7 dpi > Pre
3-Hydroxyanthranilic acid	C00632	3.948	0.015249	5 dpi > 3 dpi; 9 dpi > 3 dpi; 5 dpi > 7 dpi; 9 dpi > 7 dpi
Citramalic acid	C00815	3.744	0.019342	14 dpi > 3 dpi; 14 dpi > 5 dpi; 14 dpi > 7 dpi; 14 dpi > 9 dpi; 14 dpi > Pre
D-Galactosamine	C02262	3.697	0.019931	14 dpi > 3 dpi; 14 dpi > 5 dpi; 14 dpi > Pre; 7 dpi > Pre; 9 dpi > Pre
L-Malic acid	C00149	3.596	0.022602	3 dpi > 14 dpi; 3 dpi > 5 dpi; 3 dpi > 9 dpi; 3 dpi > Pre; 7 dpi > Pre
N-Acetylneuraminic acid	C19910	3.415	0.029465	3 dpi > Pre; 5 dpi > Pre; 9 dpi > Pre

Succinic acid	C00042	3.371	0.029911	Pre > 5 dpi; Pre > 7 dpi; Pre > 9 dpi
m-Hydroxybenzoic acid	C00587	3.356	0.029911	Pre > 3 dpi; Pre > 7 dpi; Pre > 9 dpi
L-Sorbose	C08356	3.332	0.029934	Pre > 3 dpi; Pre > 5 dpi; Pre > 7 dpi; Pre > 9 dpi

Table S6 (*cont.*). Multivariate ANOVA analysis of a metabolomics-derived dataset of nasal wash samples collected from ferrets at several time-points.

**Metabolite feature**	**KEGG ID**	**F-statistic**	**FDR**	**Fisher's LSD**
L-Serine	C00716	3.259	0.032611	3 dpi > Pre; 5 dpi > Pre; 7 dpi > Pre
Glyceric acid	C00258	3.052	0.044086	3 dpi > 14 dpi; 5 dpi > 14 dpi; 7 dpi > 14 dpi; 9 dpi > 14 dpi; Pre > 14 dpi; Pre > 7 dpi
N-Acetyl-α-D-glucosamine 1-P	C04256	3.022	0.044086	5 dpi > 14 dpi; 9 dpi > 14 dpi; 5 dpi > Pre; 9 dpi > Pre
N-Acetyl-D-glucosamine 6-P	C00357	3.022	0.044086	5 dpi > 14 dpi; 9 dpi > 14 dpi; 5 dpi > Pre; 9 dpi > Pre

In this table, P: phosphate, Pre: Pre-infection, dpi: days post-infection

Table S7. Significant discovery metabolites observed through LC-QTOF-MS analysis

**Metabolite**	**F-statistic**	**FDR**	**Formula**	**Observed Mass**	**Theoretical mass**	**Mass error (ppm)**
2,3-Dimethylsuccinic acid	12.441	2.70E-07	C6 H10 O4	146.0577	146.058457	5.182856
(+)-Isomyristic acid	11.447	3.67E-07	C14 H28 O2	228.2089	228.209479	2.537149
Stearolic acid	11.383	3.67E-07	C18 H32 O2	280.2403	280.240779	1.709247
Monoolein	10.786	6.22E-07	C21 H40 O4	356.2925	356.293208	1.987131
3-hydroxy-hexadecanoic acid	9.7278	1.83E-06	C16 H32 O3	272.2353	272.235693	1.443604
2-Hexyldecanoic acid	6.0773	0.000285	C16 H32 O2	256.2402	256.240779	2.259594
Vanillylmandelic acid	4.5567	0.00322	C9 H10 O5	198.0548	198.053372	7.210126
Phthalic acid Mono-2-ethylhexyl Ester	3.6172	0.014693	C16 H22 O4	278.1514	278.152358	3.444156

In this table, FDR: false discovery rate.

Table S8. Pathway enrichment and impact analysis of a metabolomics-derived dataset of nasal wash samples collected from ferrets.

Metabolic pathway	Total	Expected	Hits	Pathway enrichment analysis			Pathway impact analysis			
				Raw p-value	Holm p-value	FDR	Raw p-value	Holm p-value	FDR	Impact
Phenylalanine, tyrosine and tryptophan biosynthesis	4	0.0723	2	0.0019	0.1610	0.1610	0.0018	0.15516	0.1552	0.5000
Phenylalanine metabolism	10	0.1807	2	0.0134	1	0.5630	0.0130	1	0.5440	0.6190
Butanoate metabolism	15	0.2710	2	0.0296	1	0.6210	0.0286	1	0.8004	0.0000
Citrate cycle (TCA cycle)	20	0.3613	2	0.3120	1	1	0.0489	1	0.9776	0.0769
Pentose phosphate pathway	22	0.3974	2	0.0601	1	1	0.0582	1	0.9776	0.0278
Glyoxylate and dicarboxylate metabolism	32	0.5781	2	0.4510	1	1	0.1120	1	1	0.0794
Taurine and hypotaurine metabolism	8	0.1445	1	0.1380	1	1	0.1360	1	1	0.4286
Nicotinate and nicotinamide metabolism	15	0.2710	1	0.2440	1	1	0.2402	1	1	0.0000
Glycerolipid metabolism	16	0.2890	1	0.2580	1	1	0.2541	1	1	0.0935
Starch and sucrose metabolism	18	0.3252	1	0.2850	1	1	0.2811	1	1	0.0731
Terpenoid backbone biosynthesis	18	0.3252	1	ND	ND	ND	0.2811	1	1	0.1143
Pantothenate and CoA biosynthesis	19	0.3432	1	0.2990	1	1	0.2942	1	1	0.0071
Pyruvate metabolism	22	0.3974	1	ND	ND	ND	0.3322	1	1	0.0311
Propanoate metabolism	23	0.4155	1	0.3500	1	1	0.3445	1	1	0.0000
Alanine, aspartate and glutamate metabolism	28	0.5058	1	0.4080	1	1	0.4025	1	1	0.0000
Glycine, serine and threonine metabolism	33	0.5961	1	0.4620	1	1	0.4555	1	1	0.0242
Amino sugar and nucleotide sugar metabolism	37	0.6684	1	0.0285	1	0.6210	0.4947	1	1	0.0386
Pyrimidine metabolism	39	0.7045	1	0.5200	1	1	0.5132	1	1	0.0175
Tryptophan metabolism	41	0.7407	1	0.5380	1	1	0.5311	1	1	0.0516
Primary bile acid biosynthesis	46	0.8310	1	0.5800	1	1	0.5731	1	1	0.0076
Fatty acid biosynthesis	47	0.8490	1	ND	ND	ND	0.5810	1	1	0.0000
Aminoacyl-tRNA biosynthesis	48	0.8671	1	0.5960	1	1	0.5888	1	1	0.0000
Purine metabolism	65	1.1742	1	0.7090	1	1	0.7019	1	1	0.0000

In this table, ND: Not detected

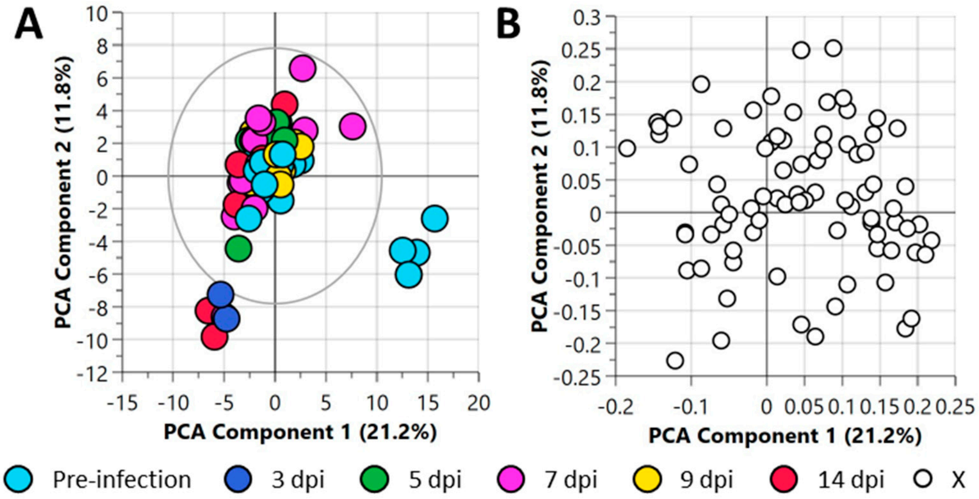

Figure S1. Principal component analysis (PCA) of the central carbon metabolism metabolite dataset of nasal wash samples collected from ferrets. **A.** PCA scatter plot, and **B.** PCA loadings plot. For this plot, R^2X (cum) = 0.525, Q^2 = 0.305. The ellipse presented in panel **A** represents Hotelling's T^2 confidence limit (95%). The colored circles in panel **A** represent each analyzed sample, while the white circles in panel **B** indicate the distribution of metabolite features between these groups.

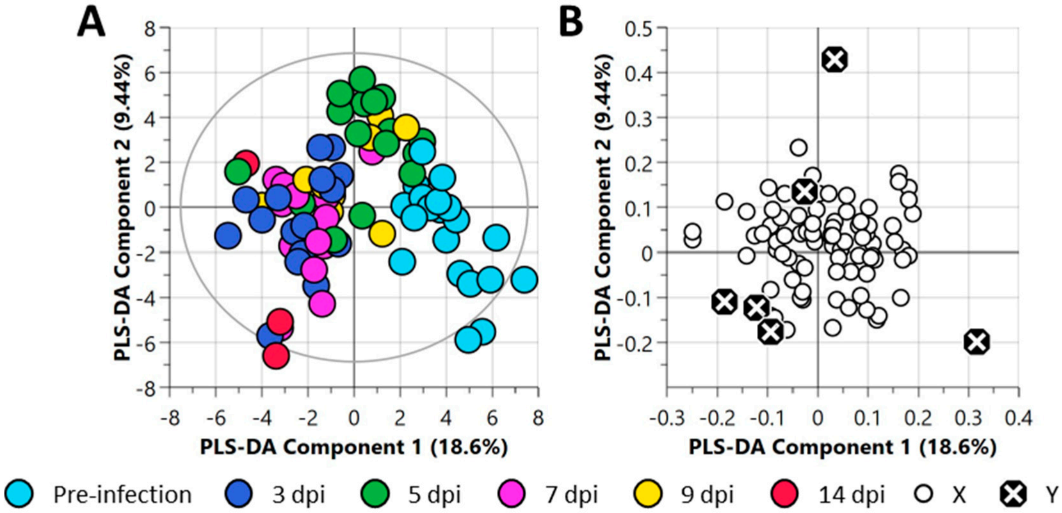

Figure S2. Partial least square discriminant analysis (PLS-DA) of the central carbon metabolism metabolite dataset of nasal wash samples collected from ferrets. **A.** PLS-DA scatter plot, and **B.** PLS-DA loadings plot. For this plot, R^2X (cum) = 0.28, R^2Y (cum) = 0.183, Q^2 = 0.0.06. The ellipse presented in panel **A** represents Hotelling's T^2 confidence limit (95%). The colored circles in panel **A** represent each analyzed sample, while the black crossed circles in panel **B** indicate the average group position for each sample cluster, with the white circles representing the distribution of metabolite features between these groups.
